# Compressing recognition network of cotton disease with spot-adaptive knowledge distillation

**DOI:** 10.3389/fpls.2024.1433543

**Published:** 2024-09-26

**Authors:** Xinwen Zhang, Quan Feng, Dongqin Zhu, Xue Liang, Jianhua Zhang

**Affiliations:** ^1^ School of Mechanical and Electrical Engineering, Gansu Agricultural University, Lanzhou, China; ^2^ Agricultural Information Institute, Chinese Academy of Agricultural Sciences, Beijing, China; ^3^ National Nanfan Research Institute, Chinese Academy of Agricultural Sciences, Sanya, China

**Keywords:** cotton diseases, deep learning, model compression, knowledge distillation, spot-adaptive

## Abstract

Deep networks play a crucial role in the recognition of agricultural diseases. However, these networks often come with numerous parameters and large sizes, posing a challenge for direct deployment on resource-limited edge computing devices for plant protection robots. To tackle this challenge for recognizing cotton diseases on the edge device, we adopt knowledge distillation to compress the big networks, aiming to reduce the number of parameters and the computational complexity of the networks. In order to get excellent performance, we conduct combined comparison experiments from three aspects: teacher network, student network and distillation algorithm. The teacher networks contain three classical convolutional neural networks, while the student networks include six lightweight networks in two categories of homogeneous and heterogeneous structures. In addition, we investigate nine distillation algorithms using spot-adaptive strategy. The results demonstrate that the combination of DenseNet40 as the teacher and ShuffleNetV2 as the student show best performance when using NST algorithm, yielding a recognition accuracy of 90.59% and reducing *FLOPs* from 0.29 G to 0.045 G. The proposed method can facilitate the lightweighting of the model for recognizing cotton diseases while maintaining high recognition accuracy and offer a practical solution for deploying deep models on edge computing devices.

## 1 Introduction

Cotton is a vital commodity in both the agriculture and textile sectors, and is an indispensable necessity for life (Feng et al., 2022). Records from the National Bureau of Statistics of China show that the cotton plantation has remained around 3.2 million hectares for the past seven years, achieving a peak output of 6.096 million tons in 2018. The diseases directly impact cotton yield and quality, with more than 80 known diseases and more than 20 frequent diseases (Li et al., 2017). To effectively prevent and control cotton diseases, it is essential to employ advanced technology for disease recognition. Currently, field-based investigations of cotton diseases rely largely on plant protection experts, which is time-consuming, labor-intensive, and suffer from poor timeliness. This method also faces difficulties in timely execution across wide areas, and the classification of disease severity is prone to subjective interference from investigators, which somewhat compromise the accuracy of disease monitoring (Shoaib et al., 2023).

With the field of computer vision is rapidly advancing, a large number of crop disease recognition and diagnosis studies have been conducted by researchers in various countries (Wani et al., 2022). The current mainstream disease recognition method is to use deep learning (Hinton and Salakhutdinov, 2006). Deep neural demonstrate excellent performance in image recognition and classification, specifically in agriculture (Ferentinos, 2018; Liu and Wang, 2021). The commonly used deep neural networks include AlexNet (Krizhevsky et al., 2012), VGGNet (Simonyan and Zisserman, 2015), ResNet (He et al., 2016), DenseNet (Huang et al., 2017), and so on.

Mohanty et al. (2016) trained a deep convolutional neural network to recognize 38 diseases of PlantVillage which is an open plant disease dataset, and found that the trained GoogleNet model achieved 99.35% accuracy, thereby establishing the method’s feasibility. Zhang et al. (2019) built a model based on AlexNet model to effectively classify and recognize six cucumber leaf diseases. Ramcharan et al. (2019) trained a CNN recognition model and used it in a mobile application. The accuracy of disease images and videos achieved 80.6% and 70.4%, respectively. Jiang et al. (2020) used the convolutional neural networks for image feature extraction of diseased rice leaves, and then applied SVM to classify and predict four rice diseases. The average correct recognition rate of the model reached 96.8%. Zeng et al. (2022) proposed the SKPSNet-50 network model to solve the problem of small and irregular early leaf spots in the maize leaf, and the recognition rate of leaf spots reached 92.90%. Tang et al. (2023) introduces the development and application of precision agriculture techniques for pest and disease control. By utilizing methods such as maize disease recognition based on HSCNN+, intelligent monitoring systems, and UAV hyperspectral remote sensing images, they have significantly enhanced the accuracy and efficiency of disease recognition and monitoring, thus promoting sustainable agricultural development. Chintalapudi et al. (2023) proposed voice biomarkers based on improved feature selection techniques for predicting Parkinson’s disease (PD). Their study analyzed voice data using Support Vector Machines (SVM) and Random Forest (RF) models, significantly improving the accuracy of PD prediction, demonstrating substantial potential in early recognition and diagnosis. Lu et al. (2023) used a modified EfficientNet to recognize healthy and diseased leaves of cotton Verticillium wilt while extracting image features, and it was found that the model achieved 93.00% accuracy in classifying healthy and diseased leaves. The aforementioned experiments all confirm that the application of convolutional neural networks to plant disease identification can effectively assist in plant disease recognition efforts.

Too et al. (2019) compared deep learning architectures such as VGG16, InceptionV4, ResNet50, ResNet101, Resnet152 and DenseNet121 based on PlantVillage. The data used for the experiment consisted of 38 plant diseases. The experimental results show that the DenseNet architecture has fewer parameters, shorter computation time, and the highest test accuracy of 99.75%. Ferentinos (2018) evaluated five CNNs-AlexNet, AlexNetOWTBn, GoogleNet, Overfeat, and VGG-using the PlantVillage dataset. According to their study, VGG emerged as the best model with an accuracy of 99.53%. Liang et al. (2019) constructed a multi-functional classification model of plant leaves based on the ResNet50 network, and estimated the plant species, disease species and disease severity respectively. The overall accuracy was 91%, 98% and 99%, respectively. Bhatt et al. (2017) compared the performance of four networks-VGG19, InceptionV3, Xception, and ResNet50-in terms of accuracy, model size, memory utilization, and inference time. Among these, ResNet50 achieved the highest accuracy of 99.7% on the tomato dataset.

VGG16, ResNet164 and DenseNet40 are very popular networks in the tasks of image classification and have been extensively studied to demonstrate high accuracy for plant disease identification.

With the increase of the parameters and complexity of neural networks, the computational and storage capabilities of the system are facing great challenges. These models can basically only run on the PCs and it is difficult to run them directly on the edge devices. In order to realize the application of deep models in the agricultural field, the models are generally compressed and deployed on the edge devices (Liu et al., 2021). Model compression technology solves the problem of model cost by reducing both the model parameters and computations. Nowadays, the mainstream model compression methods are knowledge distillation (KD), lightweight network architecture, pruning and quantization. Chen et al. (2022) proposed a model combining channel attention and channel pruning for disease identification. The model achieved 99.7% accuracy on PlantVillage and 97.7% accuracy on a local peanut leaf disease dataset. Compared to the base ResNet18 model, floating point operations (*FLOPs*) were reduced by 30.35%, parameters were reduced by 57.97%, and model size was reduced by 57.85%. Chao et al. (2021) designed a lightweight network to recognize apple leaf diseases. The network was found to have an average classification accuracy of 97.01%, which is much higher than MobileNetV1 and ShuffleNet, and has the least number of parameters. Zhu et al. (2022) compressed the cotton disease recognition model by pruning algorithm. It was found that when the pruning rate was 80%, the accuracy of all the models used was improved, and DenseNet40 had the best performance, the highest accuracy, and the lowest number of model parameters.

Knowledge distillation (Hinton et al., 2015), which serves as a prominent technique for model compression, effectively transfers the intricate knowledge encoded within the cumbersome teacher model to a more streamlined student model. This transfer is achieved by designing the student model to closely emulate the output of the teacher model, thereby ensuring maximum retention of valuable information. Based on ensuring the model’s accuracy, the size and computation load of the model are substantially reduced. Tang and Huang (2021) used tomato diseases in PlantVillage dataset as the researched object, and utilized the knowledge distillation method for training, and compared five kinds of networks such as AlexNet and VGG16. The results demonstrate that the distilled custom model exhibits remarkable accuracy in both identifying and localizing leaf disease areas, highlighting its efficacy in precision agriculture applications. The average recognition accuracy reached 97.6%, and the model size was only 4.4 M. Peng and Wang (2021) used pruning to reduce the neural network size and computational cost, and then re-trained the model through knowledge distillation to reduce the performance loss. Wang et al. (2021) proposed a DNN-based compression method using a lightweight fully connected layer to accelerate inference, pruning to remove redundant parameters, knowledge distillation to improve accuracy, and then quantization to further compress the model, which ultimately compresses the model to 0.04 Mb with an accuracy of 97.09%. Dai and Fan (2022)proposed a new network structure YOLO V5-CAcT to recognize crop diseases. Knowledge distillation is used to reduce the loss of accuracy, and then the average recognition accuracy is 94.24% by continuing to optimize the model. The model size is only 2MB, which is 88% less compared to the original model. Li and Ai (2022) used MobileNetV3 as the student model and ResNet101 as the teacher model for knowledge distillation. The accuracy on the data validation set reached 98.8%, and the model size was 23M.

In this study, they are selected as the teacher models of cotton disease recognition for knowledge distillation. Two kinds of lightweight networks, including the homogeneous and the heterogeneous networks, are selected as the student networks. The homogeneous networks with the same structure as the teacher networks include VGG8, ResNet8, and DenseNet10, while the heterogeneous networks include MobileNetV2 (Sandler et al., 2018) and ShuffleNetV2 (Ma et al., 2018). The latter two lightweight networks, are designed with a strong emphasis on improving computational efficiency and reducing runtime memory. First, we train the teacher models over the plant disease dataset. Then, in order to facilitate the knowledge transfer from a teacher model to a student model and achieve excellent classification performance, we employ spot adaptive strategy for the nine knowledge distillation algorithms. During the whole distillation process, this strategy can adaptively determine the distillation spot of a teacher model and improve the optimization efficiency. We compare the classification performances of the student models achieved from the different knowledge distillation algorithms, and try to find the optimal combination of knowledge distillation algorithm and network structure that satisfies the requirements of high accuracy, high inference speed, and small storage space, and realizes the identification of cotton diseases while satisfying the deployment situation of edge devices.

The rest of the paper is organized as follows: in Section 2, we introduce the material and methodology, including experimental data, introduction of the teacher networks used in our study, generic knowledge distillation algorithms, spot-adaptive knowledge distillation algorithms and evaluation metrics. Section 3 describes the experimental setup and results. The student networks include homogeneous and heterogeneous lightweight networks. We compare the compression effect and recognition accuracy of the teacher-student combination models with different distillation algorithms. Section 4 summarizes the work of this paper.

## 2 Materials and methods

### 2.1 Database

**Figure 1:**
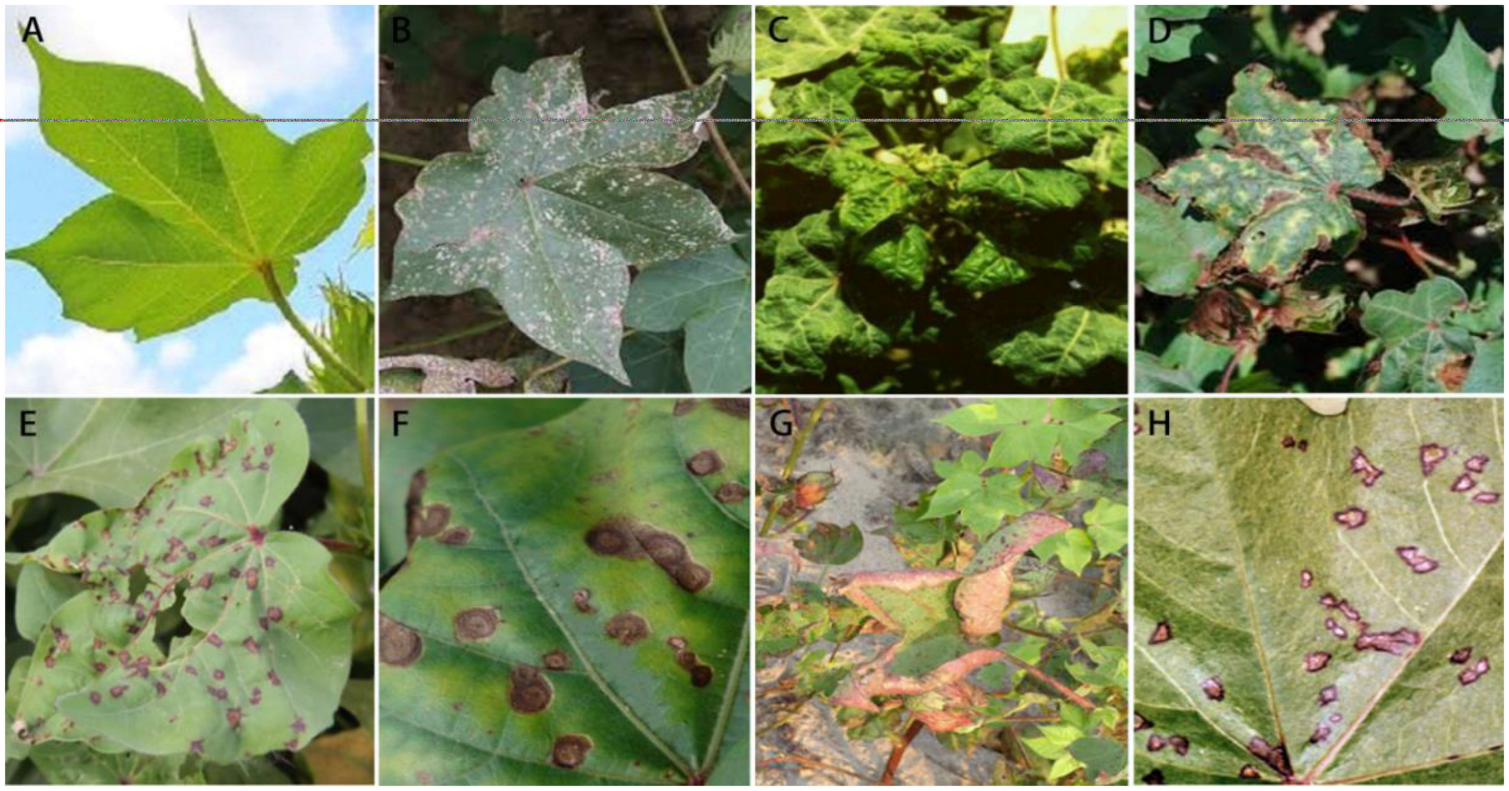
Partial images of self-built cotton disease dataset **(A)** Healthy, **(B)** Areolate mildew, **(C)** Curl virus, **(D)** Verticillium wilt, **(E)** Brown spot, **(F)** Target spot, **(G)** Fusarium wilt, **(H)** Bacterial blight.

The cotton disease dataset used in our study encompasses a diverse range of images, including those sourced from the internet as well as those captured firsthand in agricultural fields. Image acquisition is carried out using an industrial-grade camera (model MS-SUA133GC, resolution 1280×1024 pixels) and a fixed focal length lens (model FA5M06, 5 megapixels, 6 mm focal length). The images are captured from May to August over 2021-2022.This dataset contains the healthy and seven kinds of diseases and with a total of 2,151 images. The image sizes are all resized to 32×32 in the experiment. Some of the original images are shown in **Figure 1**.

**Figure 2:**
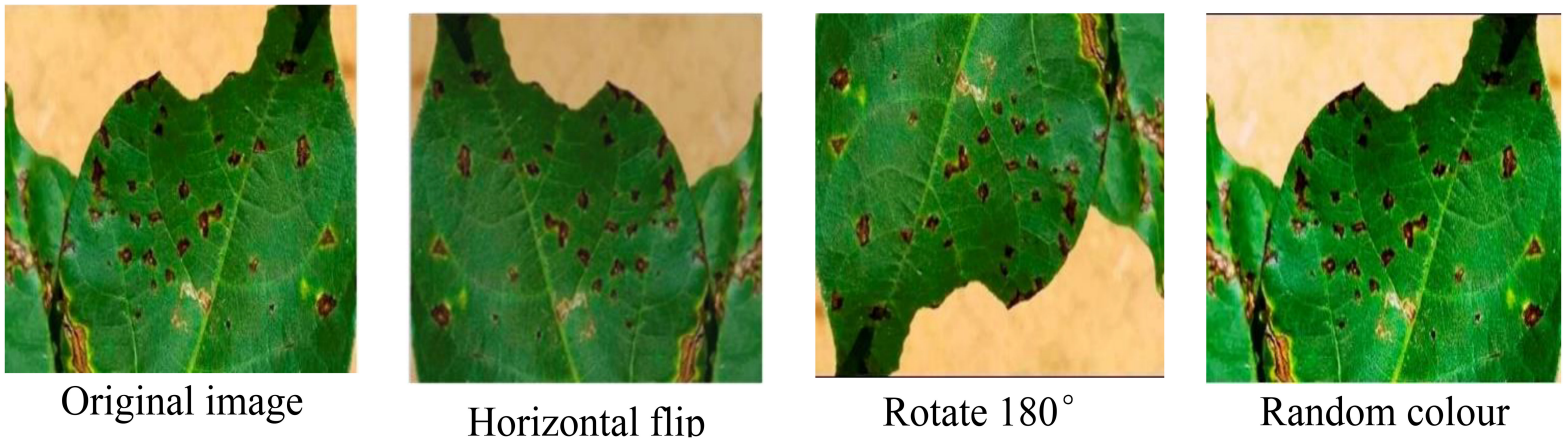
Data augmentation operations.

The self-built cotton disease dataset encompasses eight distinct categories, exhibiting the following distribution of images: 34 instances of areolate mildew, 418 cases of curl virus, 499 occurrences of bacterial blight, 264 instances of brown spot, 58 target spot samples, 419 fusarium wilt cases, 34 verticillium wilt samples, and 425 depictions of healthy leaves. It is noteworthy that the dataset does not exhibit a uniform distribution of images across these categories. Consequently, during the training phase, there exists a potential risk of the trained model exhibiting a bias towards categories that are represented by a higher number of image samples. This imbalance in the dataset’s categorical representation may have significant implications on the model’s overall performance and generalization capabilities. To solve the problem, data enhancement methods such as rotation, random color, and horizontal flip are employed to expand the number of samples of the categories with the small samples. The enhancement example is shown in **Figure 2**.

The augmented dataset comprises 170 instances of areolate mildew, 418 cases of curl virus, 499 occurrences of bacterial blight, 264 instances of brown spot, 357 target spot samples, 419 fusarium wilt cases, 170 verticillium wilt samples, and 425 depictions of healthy leaves. Subsequently, for the sake of brevity and clarity in our discussions, we shall refer to this self-constructed cotton disease dataset as SCDD (Self-built Cotton Disease Dataset).

In our experiments, the images of each category in SCDD are divided into a training set and a test set according to a ratio of 8:2, with 2,181 images in the training set and 542 images in the test set.

### 2.2 Knowledge distillation

Network Compression refers to the process of reducing the size and computational complexity of neural network models through various techniques and methods while aiming to maintain their performance. The goal of network compression is to enable deep learning models to operate more efficiently in resource-constrained environments (such as mobile devices and embedded systems), thereby reducing storage requirements, computational costs, and energy consumption.

The network compression technique employed in this study is based on spot-adaptive knowledge distillation. Knowledge Distillation is a process where a smaller neural network (referred to as the Student Model) is trained to emulate a larger neural network (referred to as the Teacher Model). The Teacher Model is characterized by its large size, computational complexity, and superior performance, while the Student Model is smaller, structurally simpler, and relatively less performant. Through this emulation process, the Student Model typically achieves comparable accuracy to the Teacher Model while significantly reducing the number of model parameters. Hence, knowledge distillation effectively compresses the model.

#### 2.2.1 Knowledge distillation algorithm

The knowledge distillation algorithm exploits the feature interpretability of teacher-based models to transform the training dataset into soft labels, simplifying the data representation and preserving important features. When training the student model, the original data is no longer used. However, the soft labels are directly used as the objective function to reduce the overfitting of the student model. The knowledge distillation algorithm can not only reduce the size of the student model but also improve the inference speed. In addition, it can improve the generalization performance of the small model to achieve higher accuracy and efficiency with limited computational resources. In this paper, a variety of knowledge distillation algorithms are used for comparative experiments in order to obtain the best performance for cotton disease recognition on the compressed model. The considered algorithms include FitNets (Romero et al., 2015), Attention Transfer (AT) (Zagoruyko and Komodakis, 2017), Neuron Selective Transfer (NST) (Huang and Wang, 2017), Probabilistic Knowledge Transfer (PKT) (Passalis and Tefas, 2018), Factor Transfer (FT) (Kim et al., 2018), Relational Knowledge Distillation (RKD) (Park et al., 2019), Similarity-Preserving (SP) (Tung and Mori, 2019), Correlation Congruence (CC) (Peng et al., 2019), and Variational Information Distillation (VID) (Ahn et al., 2019).

#### 2.2.2 Spot-adaptive knowledge distillation

**Figure 3:**
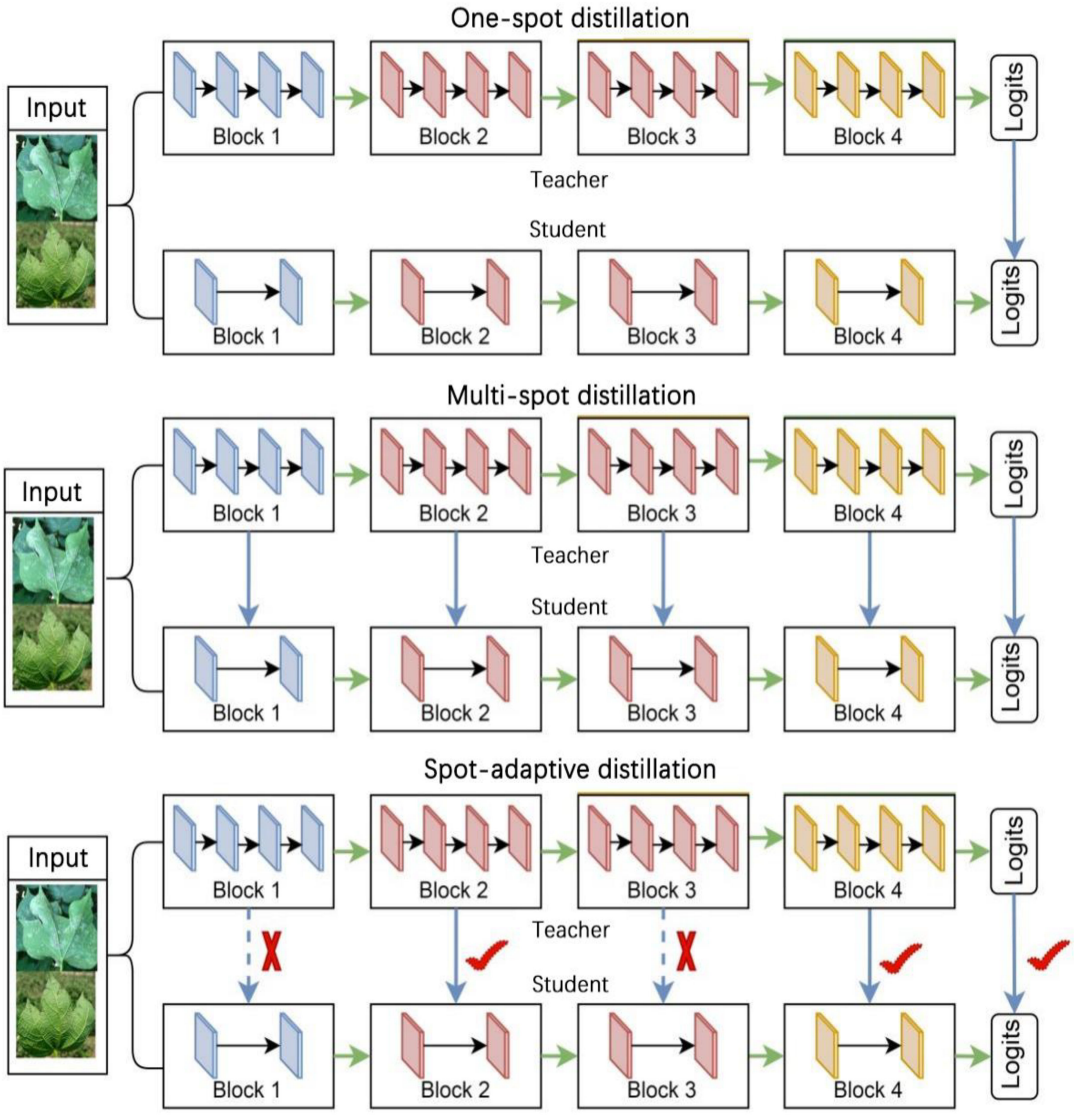
A Schematic of knowledge distillation.

Distillation strategies can be broadly categorized into one-spot distillation and multi-spot distillation based on the number of distillation spots, as shown in **Figure 3**. One-spot distillation uses only one layer in the teacher model, and multi-spot distillation is acquiring knowledge from multiple layers of the teacher network to provide more supervisory signals to the students. The multi-spot distillation method obtains more information from the teacher than one-spot distillation, so it is generally assumed that they will perform better when training student networks. Both one-spot distillation and multi-spot distillation algorithms involve human determination of distillation spots, which may lead to the problem of insufficient teacher supervision if the location of the determined spots is too sparse and over-regularization if the determined spots are too dense. To address this problem, we use a new strategy for compressing the disease identification model called spot-adaptive distillation (Song et al., 2022).

**Figure 4:**
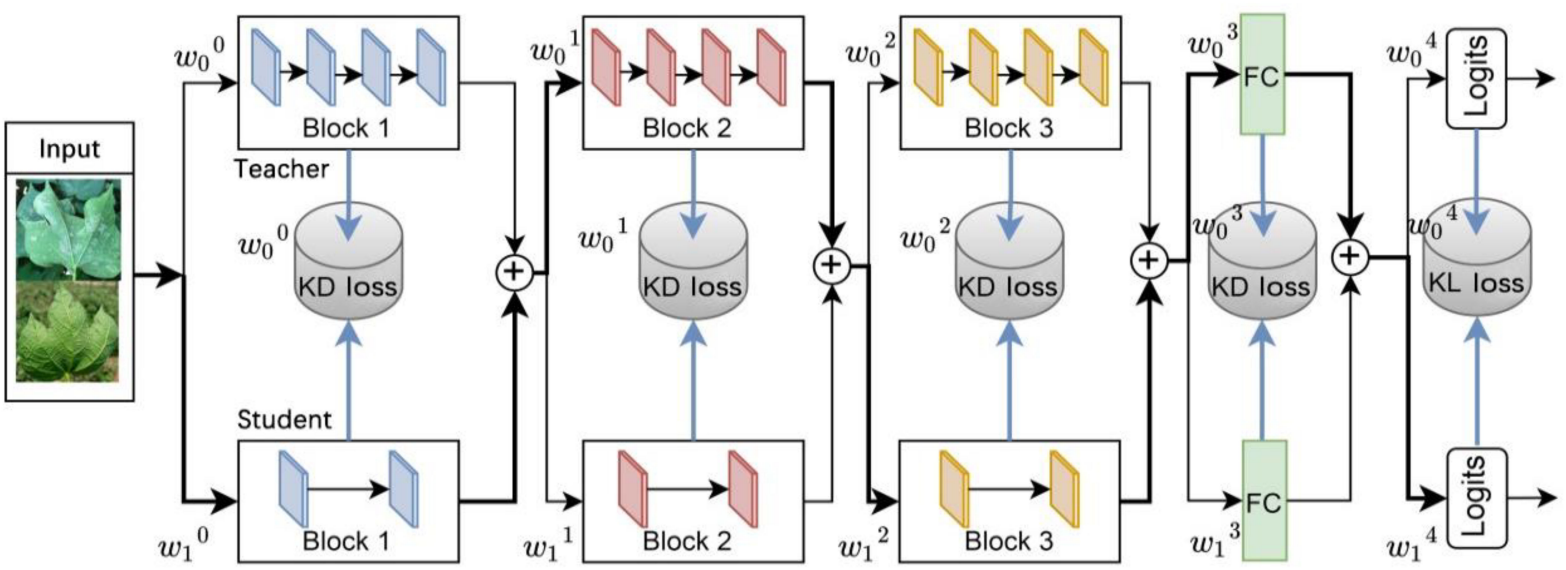
Overview of the spot adaptive knowledge distillation method.

The fundamental concept of this strategy involves the automatic determination of the distillation location and the merging of the student and teacher models into a multipath routing network. The routing network, illustrated in **Figure 4**, offers multiple paths to the output layer when data is input. Moreover, a lightweight decision network is employed to determine the optimal propagation path for each sample as it reaches a branch spot in the network. If the decision network passes the data to the layer of the teacher model, it indicates that the layer in the teacher model cannot yet be directly replaced by the corresponding layer in the student model and that the knowledge from the teacher layer needs to be distilled to the corresponding student layer. While the decision network passes data to the layers of the student model, it indicates that the layers of the student model can be directly replaced with the layers of the teacher model, yielding excellent or similar performance, and that distillation can be performed without these layers. This algorithm focuses on the location of distillation, rather than the distillation content that existing research focuses on, so it can be combined with current major distillation algorithms.

The image classification convolutional neural network typically consists of the convolutional block, fully connected layer, and softmax layer. Following the convolutional layer, there will be an activation layer and a batch normalization layer to compress the feature map. The functions of the teacher model T and student model S can usually be expressed as Formulas 1 and 2:


(1) $$\begin{array}{c} T=S\circ F^{t}\circ B_{N}^{t}\circ \cdots \circ B_{1}^{t} \end{array}$$

(2) $$\begin{array}{c} S=S\circ F^{s}\circ B_{N}^{s}\circ \cdots \circ B_{1}^{s} \end{array}$$

Where *S* denotes the softmax function, *F* denotes the linear function, $B_{i}$ represents the basis function of the *i*-th block. Superscripts *s*, *t* denote the student model and the teacher model, respectively. ∘  represents the combination operation of a function.

The multipath routing network ℳ consists of a student network S and a teacher network T . Its basic function is represented as:


(3) $$\begin{array}{c} M=S\circ \hat{F}\circ {\hat{B}}_{N}\circ \cdots \circ {\hat{B}}_{1} \end{array}$$

(4) $$\begin{array}{c} \hat{F}=wF^{t}+\left(1-w\right)F^{s} \end{array}$$

(5) $$\begin{array}{c} {\hat{B}}_{i}=w_{i}B_{i}^{t}+\left(1-w_{i}\right)B_{i}^{s},1\leq i\leq N \end{array}$$

Where *w* and $w_{i}$ are the feature fusion weights generated by the decision network, bounded by [0, 1]. When the feature fusion weights take discrete values of {0, 1}, the network turns into a combinatorial network whose layers consist of interwoven connected teacher and student layers.

The decision network consists of a lightweight, fully connected layer whose output is an *N*+1 two-dimensional routing vector, where *N*+1 denotes the number of branch spots, i.e., the number of candidate distillation spots. Each routing vector is a probability distribution from which a categorical value is randomly drawn to determine the data flow path of a branch spot in the routing network.

Spot adaptive distillation is performed by simultaneously training the routing and decision networks. The overall objective function is:


(6) $$\begin{array}{c} L=L_{student}+\beta_{1}L_{KL}+\beta_{2}L_{KD}+\beta_{3}L_{routing} \end{array}$$

Where $L_{student}$ is the cross-entropy loss between the student model goals and predictions, $L_{KL}$ is the KL scatter between the teacher model predictions and the student model predictions, $L_{KD}$ is the distillation loss of existing knowledge imposed on the intermediate layer, $L_{routing}$ is the cross-entropy loss between the goals and the routing network predictions, $\beta_{2}$ , $\beta_{1}$ and $\beta_{3}$ are hyperparameters that weigh these loss functions.

### 2.3 Teacher networks

In general, the larger the model for deep learning, the higher the accuracy of disease recognition. We use three classical large-parameter convolutional neural networks as the teacher networks, including VGG16, ResNet164, and DenseNet40, to train a high-precision disease recognition teacher model. Compared to other deep learning networks, these models have been demonstrated to be very competitive in plant disease recognition. The last layers of three networks are modified to adapt to the classification task of eight cotton diseases.

**Figure 5:**
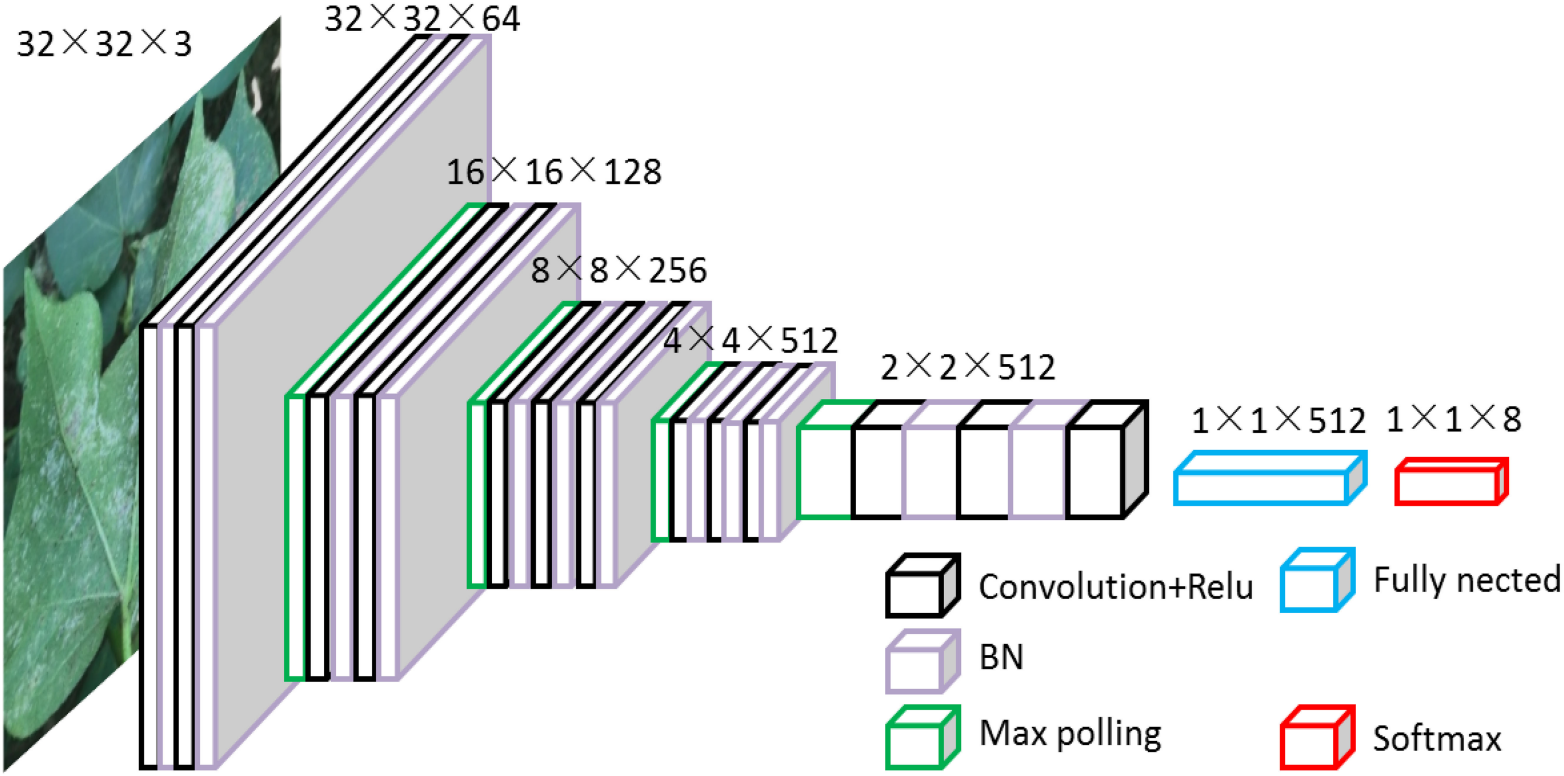
Schematic diagram of the structure of VGG.

VGG16 comprises a total of thirteen convolutional layers, three fully connected layers, and five pooling layers. The activation function used throughout is the ReLU function, exhibiting a simple structure. The convolutional and fully connected layers are often referred to as weight layers. In this network, the main responsibility of the thirteen convolutional layers and five pooling layers is feature extraction, while the three fully connected layers are dedicated to the classification task. VGG16 adopts small 3×3 convolutional kernels and 2×2 pooling kernels for all its convolutional layers. The stacking of multiple convolutional and pooling layers creates a deeper network structure. This configuration not only helps to reduce the number of parameters but also enhances the network’s fitting and representation capabilities through increased nonlinear mapping. **Figure 5** illustrates the VGG network structure. The VGG16 model utilized in this study is a modified version of the original VGG, which is smaller in size compared to the classical VGG16 model.

**Figure 6:**
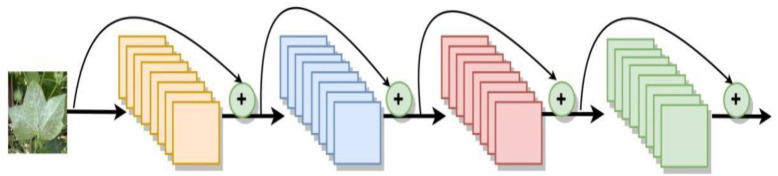
Schematic diagram of the structure of ResNet.

ResNet is a residual network formed by adding jump connections based on ordinary networks. ResNet is easier to optimize than normal networks and the performance will not decrease with increase of the network depth. ResNet introduces a residual module to solve the problem of training difficulty and slow convergence due to deeper layers. The ResNet network structure is shown in **Figure 6**. In this study, a 164-layer pre-activated pre-ResNet framework with a bottleneck structure is used.

**Figure 7:**
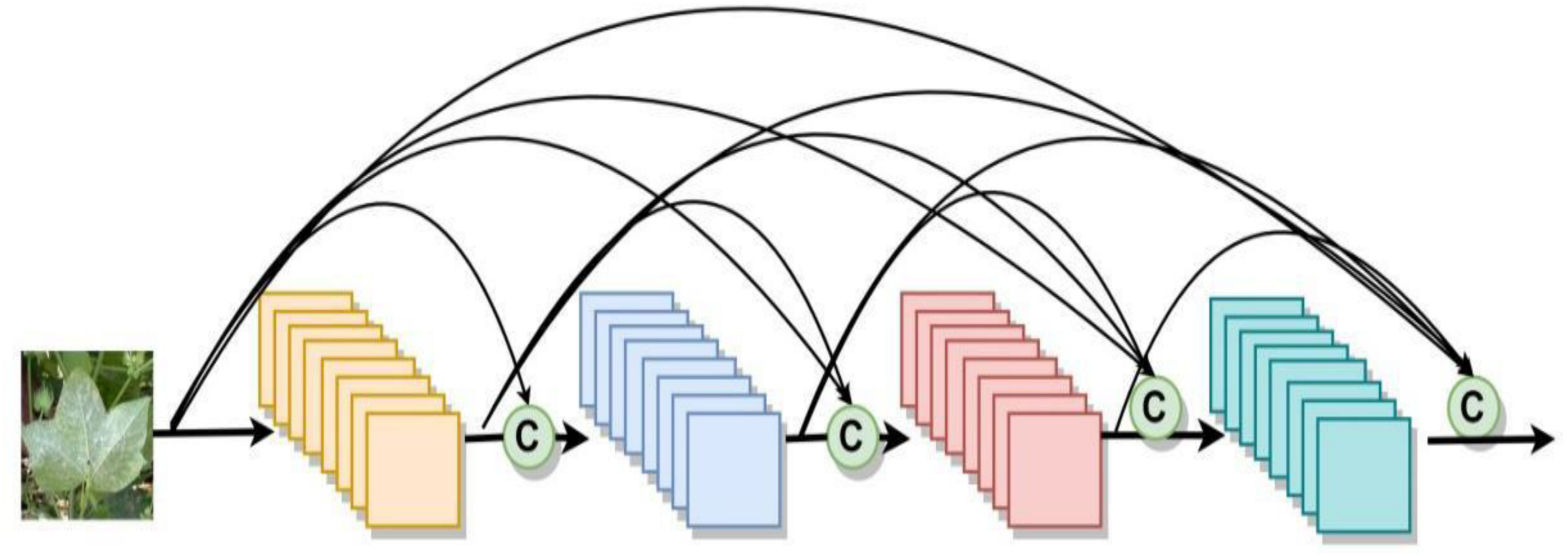
Schematic diagram of the structure of DenseNet.

DenseNet is to connect the output of each layer directly to the input of each layer behind. These inputs are not directly arithmetically summed, but spliced in feature dimensions, reducing the possibility of gradient vanishing. Furthermore, the incorporation of the bottleneck layer, translation layer, and a small growth rate serves to streamline the network architecture and minimize the number of parameters, thereby enhancing its efficiency. DenseNet has extremely high parameter utilization and shows no overfitting or accuracy degradation when increasing the number of layers. The structure of the DenseNet network is depicted in **Figure 7**. In this study, a DenseNet40 with only 40 layers is constructed.

### 2.4 Student networks

A few parameters, low complexity and fast training speed characterize student models. The operation of small network models on edge devices depends on the devices’ computing power and memory size. Some high-end edge chips’ computing power and memory size can already support certain small-scale neural networks. Zhang et al. (2021) introduced a streamlined fruit recognition algorithm tailored exclusively for edge computing devices, which has a parameter count of 5.96M, the smallest among the comparative network models, and is used in NVIDIA Jetson Xavier NX, NVIDIA Jetson TX2, and NVIDIA Jetson NANO edge devices to accomplish target recognition. Mao et al. (2023) developed an Android application RTFD-CPU, assessed the real-time growth conditions of tomatoes and strawberries. on the smartphone Redmi K30pro (Snapdragon 865 and 8 GB RAM). The size of the quantitative RTFD model is 1.33 MB. Overall, the size of the model running on edge devices is basically in the order of MB or smaller.

In the experimental phase, we designed comparative experiments for homogeneous and heterogeneous structures. A homogeneous structure refers to network models in which the layers have very similar or identical structures and configurations. However, due to the uniformity of layer structures, such models may lack flexibility and might not fully capture the diversity and complex features of the data. A heterogeneous structure refers to network models in which the layers have different structures and configurations. The advantage of this approach is that by optimizing the structure and configuration of different layers, it is possible to better capture the complex features of the data, thereby improving model performance.

MobileNetV2 and ShuffleNetV2 are classic lightweight neural networks optimized for the needs of mobile and embedded devices, offering efficient and accurate inference capabilities in resource-constrained environments. MobileNetV2, introduces depth wise separable convolution and inverted residual structures, significantly reducing computational load and parameter count. ShuffleNetV2 addresses bottlenecks in channel communication by incorporating channel shuffle and grouped convolution techniques, which significantly enhance the model’s computational efficiency and throughput. Both models exhibit substantial differences from the aforementioned teacher models in terms of design philosophy, architectural complexity, computational efficiency, and application scenarios, making them typical examples of heterogeneous structures. Thus, we select MobileNetV2 and ShuffleNetV2 as student models in the heterogeneous experiments. For the homogeneous structure experiments, we choose smaller networks with the same structure as the teacher networks, specifically VGG8, ResNet8, and DenseNet10, as the student models.

**Figure 8:**
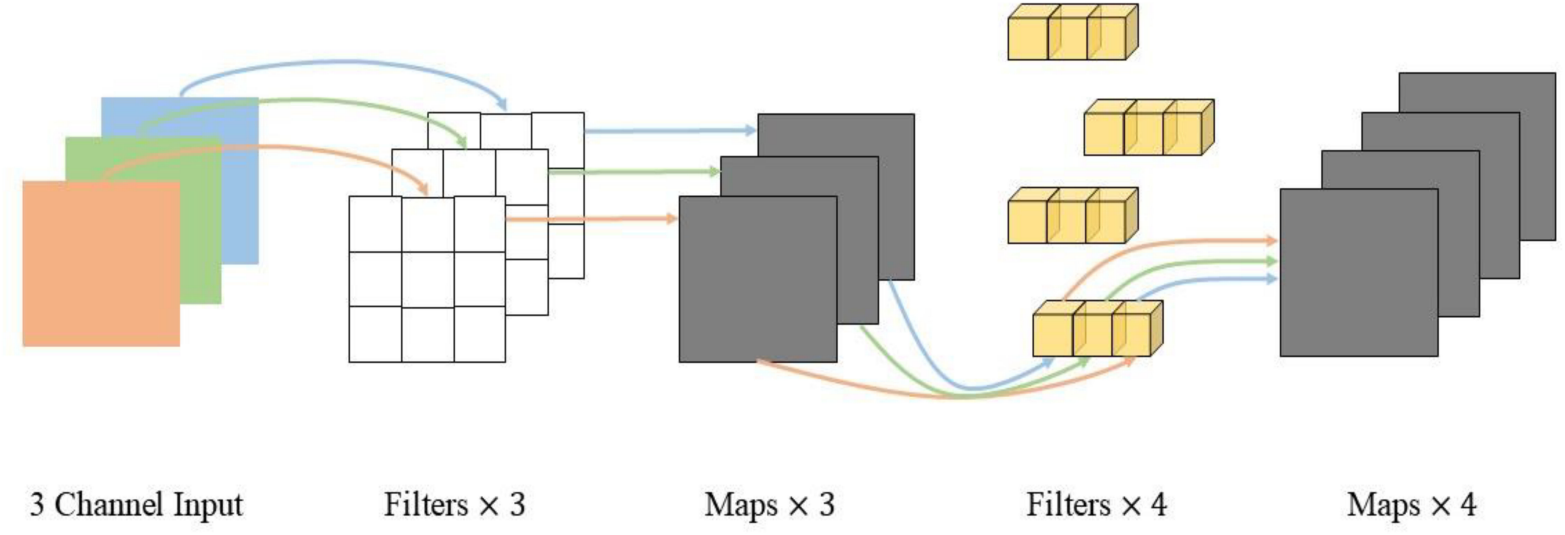
Depth wise separable convolution.

**Figure 9:**
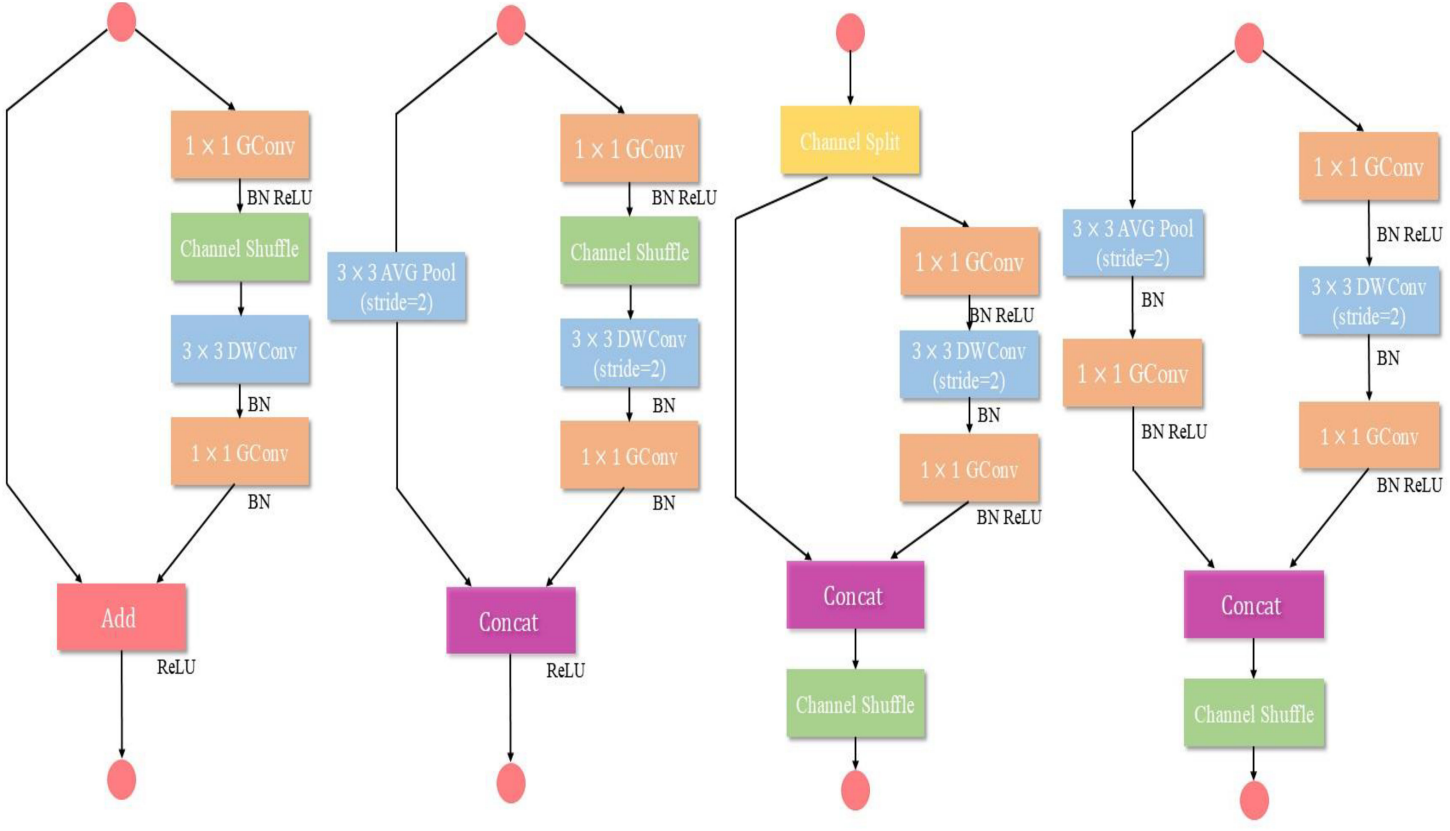
ShuffleNetV2 network architecture.

**Figure 8** illustrate depth wise separable convolution of MobileNetV2, while **Figure 9** shows the ShuffleNetV2 network structure.

### 2.5 Evaluation metrics

We evaluate the performance of the compressed models by the accuracy, the number of floating-spot operations and the model size.

The accuracy of model reflects the accuracy of model prediction. It refers to the percentage of the number of correct model predictions in the total number of data under certain experimental conditions. The formula is as follows:


(7) $$\begin{array}{c} Accuracy\;=\;\frac{TP}{TP+FP}\times 100\% \end{array}$$

Where *TP* refers to the number of correct predictions successfully made by the recognition model, and *FP* refers to the number of incorrect predictions made by the recognition model. The higher the Accuracy , the better the performance of the model.

Floating point operations ( FLOPs ) are the number of computations during the actual operation. The index used to measure the complexity of the model, and can also be interpreted as the computational workload. This value is calculated based on the depth of the model. The formula for each convolutional layer is as follows:


(8) $$\begin{array}{c} FLOPs\;=\;2HW(C_{in}k^{2}+1)C_{out} \end{array}$$

Where $C_{in}$ indicates the quantity of input channels, *k* refers to the size of the convolution kernel, *HW* refers to the height and width of the feature map. $C_{out}$ presents the number of output channels.

The calculation formula of the fully connected layer is as follows:


(9) $$\begin{array}{c} FLOPs\;=\left(2\times I-1\right)\times O \end{array}$$

Where *I* represents the quantity of input units, and *O* represents the quantity of output units.

Model size is the model’s size, independent of the size of the input image, describing the memory required. The computational resources of the edge device’s memory are extremely limited, and if the model is too complex, it cannot be loaded into the device’s memory.

To meet the application requirements of edge devices, it is essential for the compressed model to have a high classification accuracy, as well as small *FLOPs* and model size.

## 3 Experimental results and discussion

### 3.1 Experimental setup

All the settings are kept the same in distillation experiments; the batch sizes of the experiments are 64, the total epoch is 100, and the learning rate is 0.01. The learning rate is decayed by a factor of 0.1 at the 50th, 70th, and 90th epoch, and the temperature value is set to 4. The hyperparameter sum in Fml. (6) is set to 1, based on the distillation method setup. The development environment consists of the following components: the operating system is Ubuntu 18.04.6 LTS 64-bit, the programming language is Python 3.7, the deep learning framework is PyTorch 2.0.0, and the integrated development environment is PyCharm 2020.1.5. The hardware of the computer used for training is configured as follows: an Intel^®^ I7 12700KF CPU @ 2.10GHz x64 processor, 64GB RAM, and an NVIDIA RTX 3090. In the experiments, the spot-adaptive strategy and nine typical distillation algorithms is used to compress the VGG16, ResNet164, and DenseNet40 cotton disease recognition models, and the optimal compression model is selected through comparative experiments.

### 3.2 Results and discussions

First, we train all the networks and evaluate their accuracy over SCDD, as a baseline to compare the performance with the compression model after compression. The results are shown in the **Table 1**. The knowledge of VGG16, ResNet164, and DenseNet40 models is transferred in the student model using nine knowledge distillation algorithms, including FitNets, AT, SP, CC, VID, RKD, PKT, FT and NST with spot-adaptive strategy. These algorithms are combined with the original KD algorithm; the KL divergence of soft labels between teachers and students is added to improve performance. In the case of the heterogeneous student model, we investigate the accuracy of the teacher-student combinations, including VGG16-MobileNetV2, ResNet164-ShuffleNetV2, and DenseNet40-ShuffleNetV2. The experiment results are presented in **Table 2**. When the homogeneous small network is used as the student network, the teacher-student combinations are VGG16-VGG8, ResNet164-ResNet8, and DenseNet40-DenseNet10. The experimental results are shown in **Table 3**. In order to see the compression effect for the homogeneous and heteromorphic student models, based on the experimental results above, **Table 4** compares the six pairs of teacher and student networks under the NST algorithm in terms of accuracy, model size, and *FLOPs*.

**Table 1:** Baseline performance over SCDD.

	Teacher			Student(baseline)				
	VGG16	ResNet164	DenseNet40	MobileNetV2	ShuffleNetV2	VGG8	ResNet8	DenseNet10
accuracy	90.77%	88.00%	90.59%	78.41%	82.47%	89.48%	82.66%	77.12%

**Table 2:** Results of heterogeneous student models.

	VGG16-MobileNetV2	ResNet164-ShuffleNetV2	DenseNet40-ShuffleNetV2
Teacher	90.77%	88.00%	90.59%
Student	78.41%	82.47%	82.47%
Fitnets	79.15%	85.61%	85.98%
AT	83.21%	85.98%	88.38%
SP	38.56%	78.78%	82.66%
CC	81.36%	86.61%	87.08%
VID	81.92%	87.27%	86.90%
RKD	83.03%	89.30%	*90.41%*
PKT	56.27%	81.18%	72.88%
FT	80.81%	84.50%	88.74%
NST	84.32%	87.27%	**90.59%**

The bold values indicate the highest accuracy achieved under the same structure in the experiment.

**Table 3:** Results of homogeneous student models.

	VGG16-VGG8	ResNet164-ResNet8	DenseNet40-DenseNet10
Teacher	90.77%	88.00%	90.59%
Student	89.48%	82.66%	77.12%
Fitnets	90.41%	84.50%	79.15%
AT	90.27%	84.31%	84.50%
SP	66.60%	73.62%	72.14%
CC	81.33%	76.01%	80.81%
VID	90.41%	80.81%	81.92%
RKD	**90.54%**	83.95%	84.32%
PKT	67.16%	64.02%	61.99%
FT	89.67%	82.84%	81.18%
NST	*90.42%*	85.05%	84.87%

The bold values indicate the highest accuracy achieved under the same structure in the experiment.

**Table 4:** Comprehensive performance of NST.

Teacher	Student	Accuracy/%		*FLOPs*/G		Model size/MB	
		Before distillation	After distillation	Before distillation	After distillation	Before distillation	After distillation
VGG16	MobileNetV2	90.77%	84.32%	0.31	0.023	117.8	18.1
	VGG8		90.42%		0.096		31.4
ResNet164	ShuffleNetV2	88.00%	87.27%	0.26	0.045	14.0	10.2
	ResNet8		85.05%		0.013		0.63
DenseNet40	ShuffleNetV2	90.59%	90.59%	0.29	0.045	8.7	10.2
	DenseNet10		84.87%		0.015		0.37

**Figure 10:**
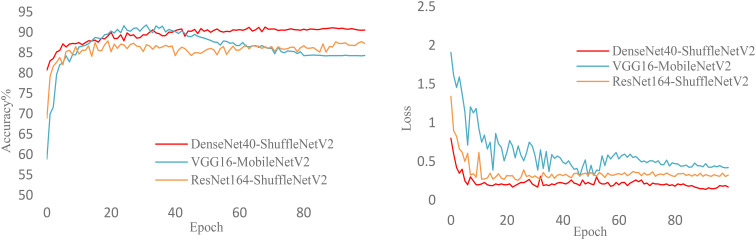
Loss and accuracy curves of heterogeneous networks (NST).

**Figure 10** illustrates the training process of various heterogeneous network models during knowledge distillation under the NST algorithm. The figure clearly shows the accuracy and loss curves for VGG16-MobileNetV2, ResNet164-ShuffleNetV2, and DenseNet40-ShuffleNetV2 as they change with epochs. It is evident that DenseNet40-ShuffleNetV2 exhibits better accuracy and lower loss. Additionally, DenseNet40-ShuffleNetV2 demonstrates more stable training and stronger robustness throughout the process.

**Figure 11:**
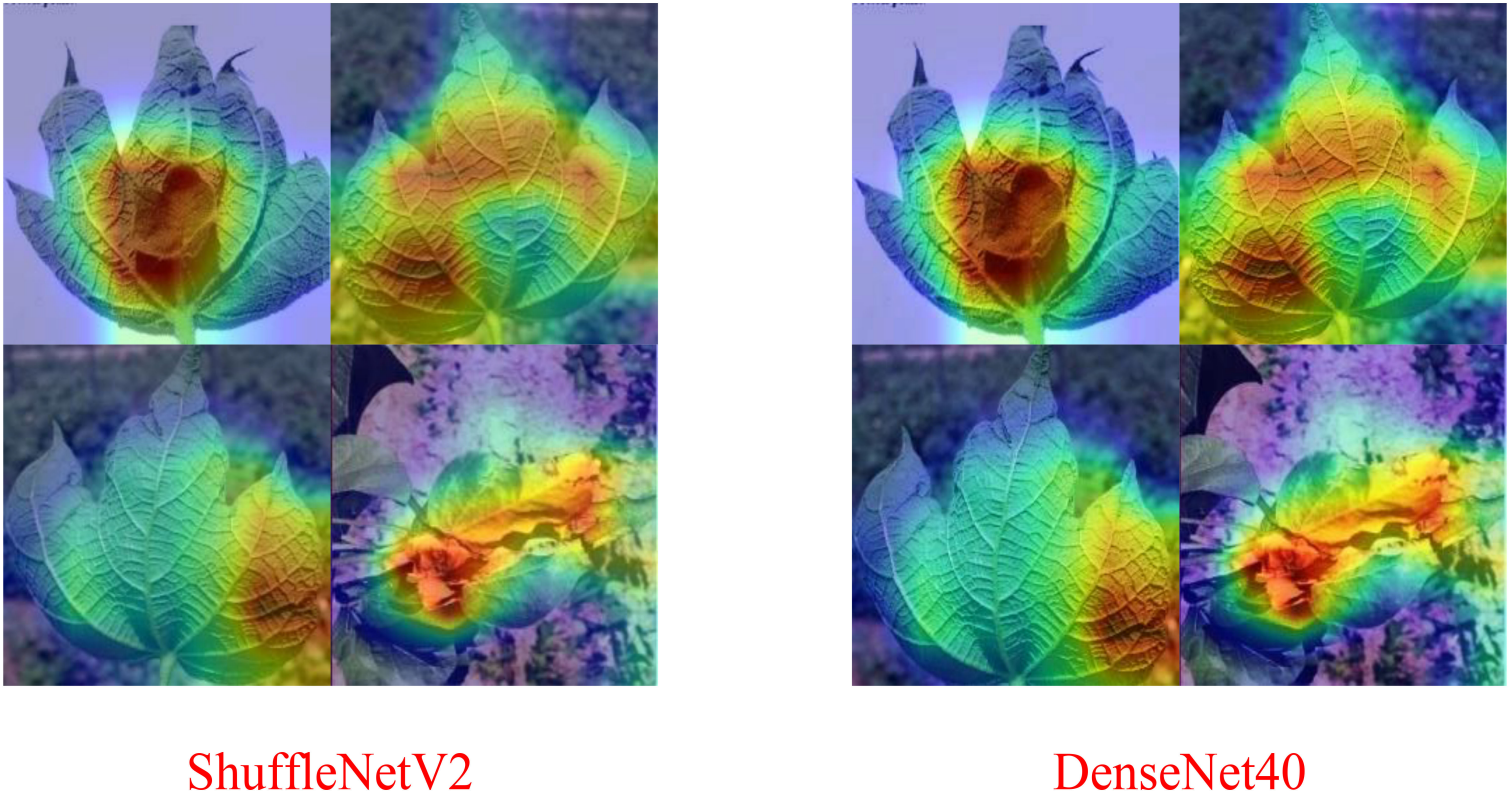
DenseNet40-ShuffleNetV2 CAM visualization (NST).

Comparison with the **Table 1**, the results of the **Table 2** reveals that when the spot-adaptive distillation algorithm is used, the heterogeneous lightweight networks exceed their respective baseline accuracies after distillation for most distillation methods, except for SP and PKT. This suggests that our scheme can broadly transfer helpful knowledge from the teacher model to the student model and improve the accuracy of the student model. For DenseNet40-ShuffleNetV2 combination, after distillation by the NST algorithm, ShuffleNetV2 had the highest recognition accuracy for cotton diseases, which increase from 82.47% to 90.59%. This accuracy is also the same as that of DenseNet40 as a teacher network, without losing any accuracy of the teacher network. As far as the distillation algorithms are concerned, the combined spot-adaptive RKD and NST maintain high accuracy for various teacher/student model combinations, with average accuracies of 87.58% and 87.39%, respectively. It shows that their distillation results have good robustness. As shown in **Figure 11**, the Gradient-weighted Class Activation Map (CAM) demonstrates the recognition effect of DenseNet40 and ShuffleNetV2 on the same cotton disease leaf image. It can be observed that there is almost no difference in the recognition effect between the teacher model and the student model. This demonstrates that knowledge from the teacher network is well transferred to the student network.

**Figure 12:**
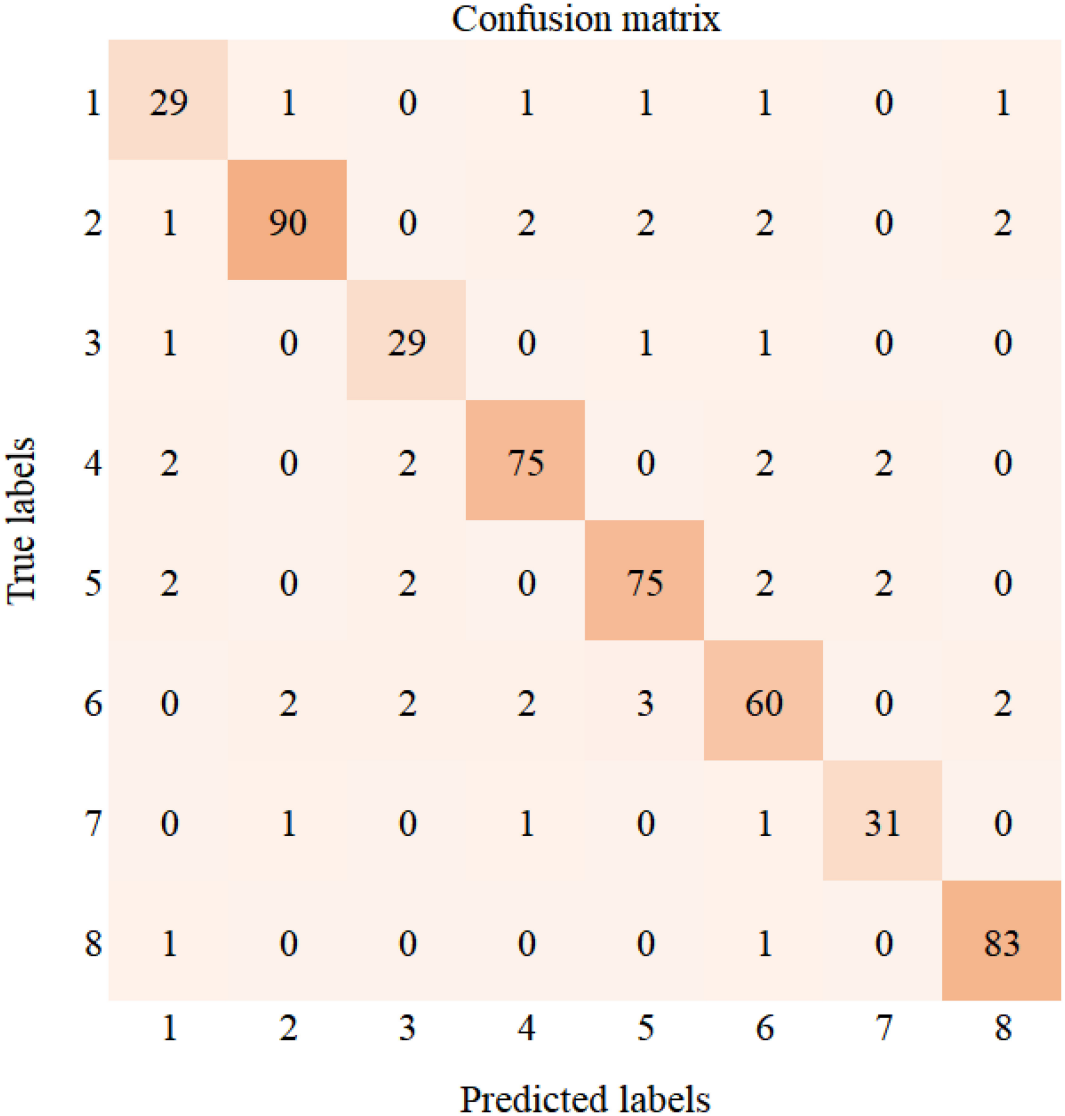
Confusion matrix of DenseNet40-ShuffleNetV2 (NST).

To further analyze the performance of the DenseNet40-ShuffleNetV2 model distilled using the NST algorithm, **Figure 12** presents the confusion matrix of this model on the cotton validation set. The values on the diagonal represent the number of correctly predicted samples. The validation set of the cotton dataset contains a total of 521 samples. The categories from Type 1 to Type 8 correspond to areolate mildew (34 samples), bacterial blight (99 samples), brown spot (32 samples), curl virus (83 samples), fusarium wilt (83 samples), target spot (71 samples), verticillium wilt (34 samples), and healthy leaves (85 samples), respectively. The confusion matrix illustrates the model’s recognition capability on the validation set. From the confusion matrix, we can observe that the model’s ability to recognize areolate mildew needs improvement. Target spot is the most frequently confused disease. Meanwhile, the model demonstrates strong recognition capabilities for most of the diseases.

**Figure 13:**
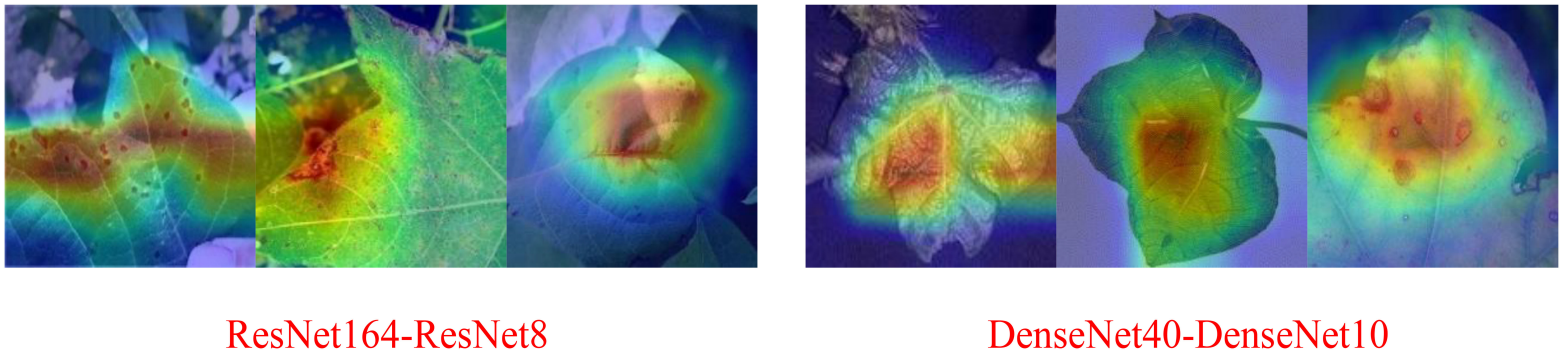
ResNet164-ResNet8 and DenseNet40-DenseNet10 CAM visualization (NST).

As shown in the **Table 3**, in terms of the robustness of the distillation algorithm, NST and RKD still perform better. Under the three teacher-student combinations, the average accuracies of the student models are 86.78% and 86.27%, respectively, ranking the top two. Comparing **Tables 1**, **3**, the NST algorithm achieves the best distillation results for both the ResNet164-ResNet8 and DenseNet40-DenseNet10 combinations, which show a significant improvement in the recognition accuracy compared to the baseline. Only at VGG16-VGG8 is the distillation effect of NST ranked second, but the accuracy after distillation differs from the first method by 0.12%. **Figure 13** shows the CAM images of ResNet164-ResNet8 and DenseNet40-DenseNet10 based on the NST algorithm, clearly highlighting the regions of interest for the models.

As can be seen from **Tables 2**, **3**, except in a few cases, there is a slight decrease in the average accuracy when using the homogeneous small network as the student model than when distilling with the heterogeneous lightweight network as the student model. Overall, among the nine knowledge distillation algorithms that employ spot-adaptation, the NST distillation algorithm do better than others. The results in the **Table 4** show that under the NST algorithm, when the heterogeneous lightweight network is used as the student network, the distilled ShuffleNetV2 has a smaller model size and higher accuracy than the MobileNetV2, but the *FLOPs* are slightly larger. When DenseNet40 is used as a teacher model to transfer the knowledge to ShuffleNetV2, the highest accuracy is achieved, and compressing 84.48% of the *FLOPs*. For the homogeneous student network, the compression effect is very remarkable. Especially the ResNet8 and DenseNet10 networks, the size of the former is compressed by 95.32% and FLOP by 95%, and the size of the latter by 96.26% and *FLOPs* by 94.83%. For VGG8, the accuracy is best among three homogeneous networks. However, it only compresses 73.44% of size and 69.03% of *FLOPs*. Therefore, VGG8 has no advantage over the other homogeneous student models.

In a comprehensive comparison, when DenseNet40 is used as a teacher model to transfer the knowledge to ShuffleNetV2, the NST algorithm with added adaptivity shows strong performance over the test dataset. Meeting the requirements of high accuracy, high inference speed, and low storage space. We consider this model to be the most appropriate when being deployed on the edge device of a plant protection robot.

## 4 Conclusion

Deep convolutional neural network is a mainstream method used for plant disease recognition. However, difficulties arise when deployed in the edge devices due to their significant model parameters and amount of calculation. In order to solve the problem of plant protection robots identifying cotton diseases in the field, we utilize the method of knowledge distillation to compress the network. We first select VGG16, ResNet164, and DenseNet40 to train the cotton disease recognition model and use them as teacher models. The teacher model is then distilled to the student model using nine typical distillation algorithms guided by a spot-adaptive strategy. We investigate two kinds of the student model, namely heterogeneous and homogeneous lightweight network. The former include ShuffleNetV2 and MobileNetV2, while the latter include VGG8, ResNet8 and DenseNet10. Experimental results show that, in most cases, the distillation algorithms with spot-adaptive strategy improve the accuracy of the student model compared with the baseline. Among them, NST and RKD have the best robustness for various teacher-student combinations. When distilling knowledge via NST, DenseNet40-ShuffleNetV2 achieves the best comprehensive performance. The accuracy of ShuffleNetV2 after distillation is increased from 82.47% to 90.59% and the *FLOPs* decreased by 84.48%. We use DenseNet40 as the teacher network, ShuffleNetV2, which is distilled by NST algorithm, as the disease recognition model, and deploy the model on the edge device of the developed plant protection robot.

In this paper we focus on knowledge distillation for CNN networks. In recent years transformer networks have been shown to have higher image classification accuracy while the complexity of the structure is much higher than that of CNNs. In the future we will investigate the compression of transformer networks to improve the accuracy of disease recognition.

## Data Availability

The raw data supporting the conclusions of this article will be made available by the authors, without undue reservation.
